# DHX34 and NBAS form part of an autoregulatory NMD circuit that regulates endogenous RNA targets in human cells, zebrafish and *Caenorhabditis elegans*

**DOI:** 10.1093/nar/gkt585

**Published:** 2013-07-04

**Authors:** Dasa Longman, Nele Hug, Marianne Keith, Corina Anastasaki, E. Elizabeth Patton, Graeme Grimes, Javier F. Cáceres

**Affiliations:** MRC Human Genetics Unit, Institute of Genetics and Molecular Medicine, University of Edinburgh, Western General Hospital, Edinburgh EH4 2XU, UK

## Abstract

The nonsense-mediated mRNA decay (NMD) pathway selectively degrades mRNAs harboring premature termination codons but also regulates the abundance of cellular RNAs. We sought to identify transcripts that are regulated by two novel NMD factors, DHX34 and neuroblastoma amplified sequence (NBAS), which were identified in a genome-wide RNA interference screen in *Caenorhabditis elegans* and later shown to mediate NMD in vertebrates. We performed microarray expression profile analysis in human cells, zebrafish embryos and *C. elegans* that were individually depleted of these factors. Our analysis revealed that a significant proportion of genes are co-regulated by DHX34, NBAS and core NMD factors in these three organisms. Further analysis indicates that NMD modulates cellular stress response pathways and membrane trafficking across species*.* Interestingly, transcripts encoding different NMD factors were sensitive to DHX34 and NBAS depletion, suggesting that these factors participate in a conserved NMD negative feedback regulatory loop, as was recently described for core NMD factors. In summary, we find that DHX34 and NBAS act in concert with core NMD factors to co-regulate a large number of endogenous RNA targets. Furthermore, the conservation of a mechanism to tightly control NMD homeostasis across different species highlights the importance of the NMD response in the control of gene expression.

## INTRODUCTION

The nonsense-mediated mRNA decay (NMD) is a highly conserved surveillance pathway that targets mRNAs harboring premature termination codons (PTCs) for degradation and also regulates the expression of naturally occurring transcripts (1–3). It can also act to modulate the phenotypic outcome of several genetic disorders that are caused by nonsense mutations or frameshifts that generate PTCs (4). As such, it has an important role in the control of gene expression.

The core NMD factors were originally identified using genetic screens in *Saccharomyces cerevisae* and in *Caenorhabditis elegans*. In nematodes, seven genes that are essential for NMD were identified and termed, *smg-1-7* (for suppressor with morphological effect on genitalia), as mutations of these genes causes abnormal morphogenesis of the male bursa and the hermaphrodite vulva besides their effect on NMD (5,6). There are three genes in *S. cerevisae*, up-frameshift 1–3 (UPF1–3), which are orthologues of *C. elegans smg-2*, *smg-3* and *smg-4* genes (7,8). Subsequently, orthologues of all these genes were found in several species, including *Drosophila* and mammals [for a review see (9)].

The ATP-dependent RNA helicase, UPF1/SMG2 is a central NMD factor, which undergoes cycles of phosphorylation and dephosphorylation that are essential for NMD to proceed. The UPF1 kinase complex SMG1c, comprising the protein kinase SMG1, a phosphoinositide 3-kinase-like kinase and two additional subunits, SMG8 and SMG9, phosphorylates UPF1 at multiple [S/T]Q motifs (10–13). The remaining SMG genes act to modify the state of UPF1/SMG2 by promoting its phosphorylation (SMG3 and SMG4) or facilitating its dephosphorylation (SMG5, SMG6 and SMG7) that also requires the catalytic and structural subunits of protein phosphatase 2 A (PP2A) [(14,15), reviewed by (16)]. The exon junction complex (EJC) is a multiprotein complex that assembles on exon junctions as a consequence of pre-mRNA splicing and recruits factors required for mRNA transport, translation, NMD and mRNA localization (17–20) [reviewed by (21)]. Premature translation termination at a PTC leaves downstream one or more EJC complexes that are not removed from the mRNA and act to recruit the NMD machinery. The EJC core anchors the UPF proteins (UPF1–3) to mRNA, and UPF2 binding to the N-terminal domain of UPF1 results in a large conformational change leading to the activation of the UPF1 helicase activity (22–24). The recognition of PTCs involves the assembly of a complex comprising the NMD factors SMG1 and UPF1 and the translation release factors eRF1 and eRF3, termed SURF. In a subsequent step, the SURF complex interacts with the EJC to form the decay-inducing complex that triggers UPF1 phosphorylation and dissociation of eRF1 and eRF3. As a result, phosphorylated UPF1 recruits additional NMD factors (SMG5/7 and SMG6), and further rearrangements of this complex lead to mRNA degradation (25–27). SMG6 is recruited to the EJC via two conserved EJC-binding motifs (28) and is the endonuclease that initiates cleavage in the vicinity of the PTC in both *Drosophila* and in humans (29,30). Phosphorylated UPF1 also acts as a platform to recruit PNRC2, human proline-rich nuclear receptor coregulatory protein 2, providing a link with the decapping activator, DCP1a (31).

A genome-wide RNA interference (RNAi) screen in *C. elegans* resulted in the identification of two novel NMD factors, termed *smgl-1* and *smgl-2*, that unlike *smg* genes are essential for viability (32). Both genes are highly conserved throughout evolution and have clear orthologues in mouse, human and fugu, but they are absent in yeast. The *C. elegans* gene *smgl-1* corresponds to human neuroblastoma amplified sequence (NBAS), also known as neuroblastoma amplified gene (NAG), which was found to be amplified in human neuroblastomas (33,34). The human homologue of the *C. elegans smgl-2* gene is the DExH/D box protein, DHX34 (DEAH box protein 34). Although the role of this helicase is not known, DExH/D helicases are commonly involved in many aspects of RNA metabolism including transcription, pre-mRNA splicing, translation and mRNA decay (35,36). We showed that both proteins act in the NMD pathway both in human cells and also in zebrafish (32,37). Morpholino-induced depletion of zebrafish Dhx34 and Nbas proteins resulted in severe developmental defects and reduced embryonic viability. Moreover, Dhx34 and Nbas are required for degradation of PTC-containing mRNAs in zebrafish embryos. The phenotypes observed in both Dhx34 and Nbas morphants are similar to defects in Upf1, Smg-5 or Smg-6- depleted embryos (38), suggesting that these factors affect the same pathway and confirming that zebrafish embryogenesis requires an active NMD pathway (37).

Importantly, the NMD pathway not only degrades PTC-containing mRNAs but is also involved in global regulation of gene expression. Studies involving expression profiles in *S. cerevisae*, *Drosophila melanogaster* and human cells lacking individual NMD factors revealed that ∼10% of naturally occurring transcripts are regulated by NMD (39–42). This suggests that the NMD process has to be tightly regulated to ensure proper gene expression. Indeed, a negative feedback regulatory network that maintains a tight control on the levels of seven NMD core factors has been recently reported in mammals. This regulatory mechanism presumably operates across all different branches of the NMD pathway in a cell-type an/or developmentally regulated manner and allows cells to buffer environmental stresses by regulating the NMD response (43,44).

Here, we identify target genes regulated by the NMD factors, DHX34 and NBAS, in human cells in culture, in the zebrafish *Danio rerio* and in the nematode *C. elegans.* We sought to address whether these two novel NMD factors co-regulate cellular targets with the core NMD machinery and are general effectors of the NMD pathway in different organisms. Indeed, a large co-regulation of targets between DHX34 and NBAS and core NMD factors was observed. We also show that these novel NMD factors predominantly regulate the expression of genes involved in the cellular stress response in all three species. We also present evidence that both DHX34 and NBAS participate in a negative regulatory feedback mechanism that was previously described for core NMD factors in mammalian cells. Importantly, we show that this regulatory mechanism is also present in zebrafish and in *C. elegans*, strongly suggesting that a tight control of the NMD response is required throughout evolution. We propose that the NMD process is part of a conserved mechanism that helps the organism to respond to cellular and environmental stress.

## MATERIALS AND METHODS

### Cell culture and shRNA-mediated depletion

HeLa cells were grown in Dulbecco’s modified Eagle’s medium (Invitrogen) supplemented with 10% fetal calf serum and incubated at 37°C in the presence of 5% CO2. For shRNA-mediated depletions, Hela cells were transfected with pSuper (puro) plasmid containing gene-specific shRNAs against UPF1 and UPF2 that were previously described (42,45) and were transfected using Lipofectamine 2000 following manufacturer’s instructions. The sequences of shRNAs are included as follows: shNAG: 5′-GCTATGACCTACTAGAATG-3′; shDHX34: 5′GAGCATCGACTGTACGAAA-3′; shUPF1: 5′-GATGCAGTTCCGCTCCATT-3′; shUPF2: 5′-GAAGTTGGTACGGGCACTC-3′; shSMG1: 5′-CACTTCAGATAACTGAGAG-3′.

Transfections were performed in six-well plates in three biological replicates; cells were expanded into 10-cm dishes and selected by growth in complete media supplemented with 0.7 μg/ml puromycin for 5 days post-transfection. Before RNA isolation, cells were washed with phosphate buffered saline. Total RNA was isolated using the Qiagen RNAeasy kit following manufacturer’s instruction. The quality of RNA was assessed by Agilent 2100 Bioanalyzer using RNA 6000 Nano Kit and NanoDrop 8000 spectrophotometer readings.

### Microinjection of zebrafish embryos

Zebrafish embryos and adults were raised and maintained at 28.5°C on a 12 h light/dark cycle. Embryos were collected by pair matings of a wild-type stain (AB-TL) and were staged as described previously (46). Injections were performed on wild-type zebrafish embryos using a nitrogen-powered Picospritzer III microinjector (Intracel) linked to a Nikon SMZ 1000 stereomicroscope. One-cell stage embryos were injected with 4 nl of 250 µM translation block morpholino oligonucleotides (MOs) against *upf1* (38), *dhx34* and *nbas* (37), as well as their respective 5 bp mismatch control MOs (Gene-Tools LLC). Morpholino antisense oligonucleotide sequences are provided on Supplementary Table S5. The effect of MO-induced depletion was assessed by the appearance of an embryonic phenotype, as described previously (37,38), whereas control mismatch MOs displayed a wild-type phenotype. Control and MO-injected embryos (24 hpf) were manually dechorionated and directly homogenized in 500 µl of Trizol (Invitrogen) using a 25 G needle. Total RNA was prepared using Trizol (Invitrogen) following manufacturer’s instructions. The quality of RNA was confirmed by Agilent 2100 Bioanalyzer using RNA 6000 Nano Kit and NanoDrop 8000 spectrophotometer readings.

### *Caenorhabditis elegans* culture and RNAi

Wild-type *C. elegans* worms (N2 strain) were grown on standard Nematode Growth Medium (NGM) plates seeded with OP50 *Escherichia coli* bacteria at 20°C. For feeding-mediated RNAi, N2 worms were synchronized at L1 larval stage and grown on NMG plates containing 1 mM isopropylthio-β-galactoside (IPTG) and 50 μg/ml ampicillin. RNAi plates were seeded with bacteria carrying inducible plasmid expressing gene-specific dsRNA (47). For *smg-*2 depletion, we used clone ID Y48G8AL.6, plate 10022, well H11 from ORF RNAi library (48). For *smgl-1*and *smgl-2* depletion, we used clones ID F20G4.1, location I-3L20 and clone ID Y37E11AM.1, location IV-8k14, respectively (47). Synchronized worms were grown on RNAi plates at 20°C for 96 h. Before RNA isolation, worms were washed in M9 buffer and homogenized in Trizol (Invitrogen) using a 25 G needle. RNA was prepared following manufacturer’s instructions. The quality of RNA was assessed by Agilent 2100 Bioanalyzer using RNA 6000 Nano Kit, and NanoDrop 8000 spectrophotometer readings.

### Microarray gene expression analysis

**Figure 1.**
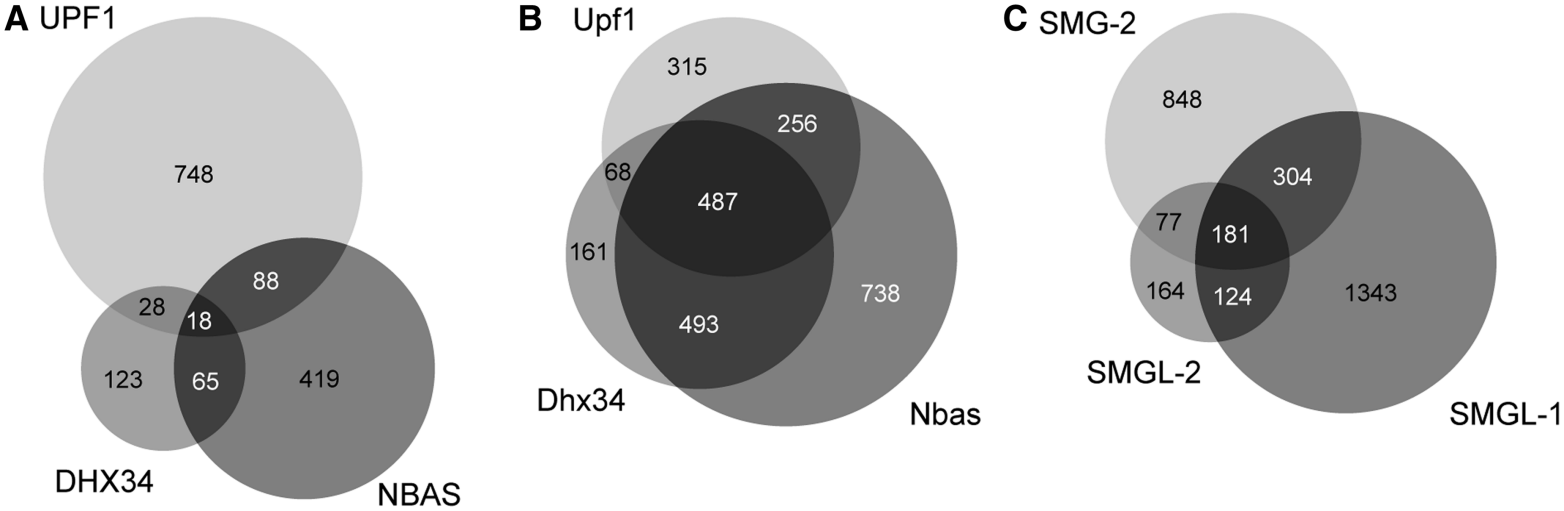
DHX34, NBAS and UPF1 regulate a common set of transcripts. Venn diagrams illustrate the overlap among genes upregulated by each of these three NMD factors in (**A**) human cells (**B**) Zebrafish and (**C**) *C. elegans*. Areas are proportional to the number of regulated genes. In each species, target coregulation is significant (*P* < 0.0001 for all individual overlaps).

**Figure 2.**
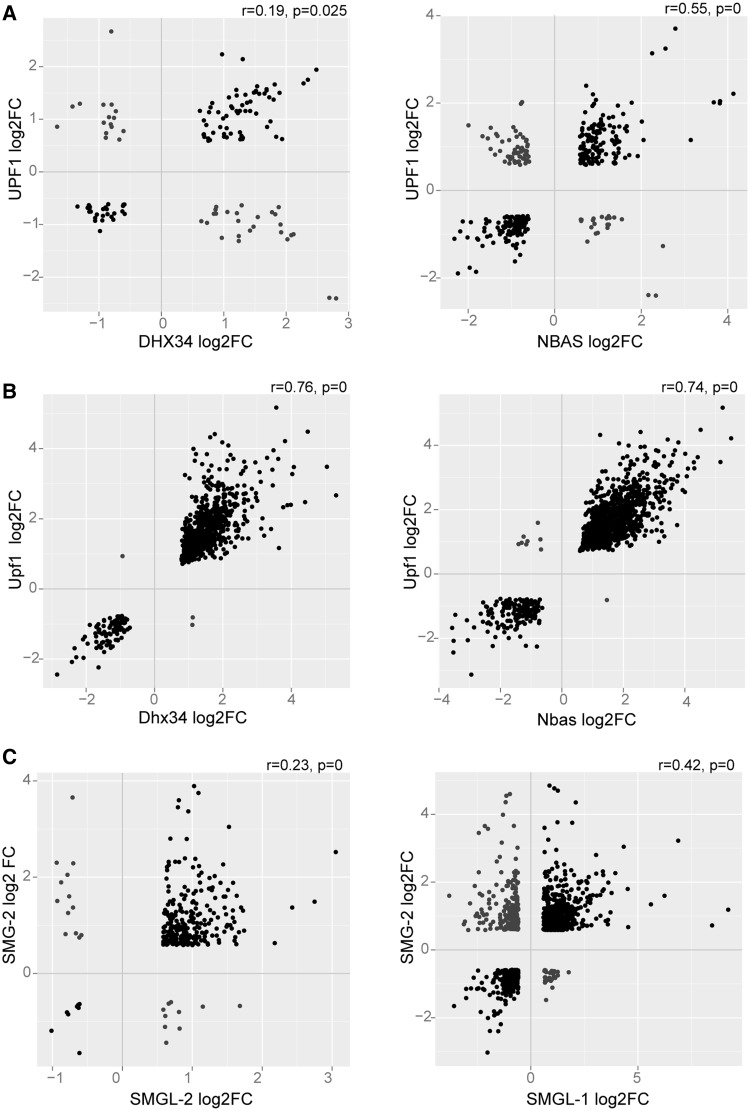
Scatter plots show a significant positive correlation between the level of regulation of target genes by DHX34 and NBAS compared with the core NMD factor, UPF1 in (**A**) human cells (**B**) Zebrafish (**C**) *C. elegans*. Each dot corresponds to a regulated gene and black dots represent coregulated genes. Spearman’s rho and *P*-values are indicated.

For microarray expression profiling, we used the NimbleGen 12 × 135 K eukaryotic arrays for human, zebrafish and *C. elegans*. For each species, RNA was prepared as described earlier in the text. In general, 10 μg of total RNA was reverse-transcribed using a SuperScript Double-Stranded cDNA Synthesis Kit (Invitrogen) following manufacturer’s instructions. Two micrograms of the resulting double-stranded cDNA was sent for further processing by the Nimblegen array service, which provided the raw and normalized microarray expression data. Before differential expression analysis, probes with Standard Error of expression >0.8 across array groups and probes with no or multiple gene annotations were removed. The R (2.14.2), Bioconductor package LIMMA was used to determine differentially expressed genes (49). The *P*-values were adjusted for multiple testing with a false-discovery rate (50). Probe sets with fold-change >1.5 and *q*-value <0.05 were used as a cut-off for human and *C. elegans* microarrays. For zebrafish microarrays, a fold-change >1.5 and *q*-value < 0.01 was considered significant. To identify significant enrichment in the overlap for differentially expressed gene sets, we used a one tailed Fisher’s exact test (Figure 1). Spearman’s rho and associated *P*-value used in Figure 2 were calculated using the R function cor.test. Gene ontology (GO) terms that were significantly overrepresented in regulated targets were identified using DAVID functional annotation tool (http://david.abcc.ncifcrf.gov). Functional categories of Sequence feature, GO term: biological process and Kyoto Encyclopedia of Genes and Genomes (KEGG) pathway were included in the analysis.

### RT-qPCR

For human samples, 2 μg of total RNA was reverse-transcribed using Transcriptor Universal cDNA Master kit (Roche) following manufacturer’s instructions. The resulting cDNA was diluted 1:10 before qPCR. Real-time PCR was performed using target-specific primers and probes pre-plated on the ready custom panel and 2× Probes Master kit (Roche) following manufacturer’s instructions. For zebrafish and *C. elegans* samples, cDNA was prepared using RT shots (Primerdesign). Two micrograms of total RNA was reverse-transcribed and resulting cDNA diluted 1:10 for subsequent qPCR analysis. Real-time PCR was performed using target-specific primers and probes (Primerdesign) and 2× Precision Mastermix (Primerdesign) following manufacturer’s instructions. Real time PCR was run on LightCycler 480 (Roche) in three technical replicates. Data analysis was performed using the LightCycler 480 software. A list of human, zebrafish and *C. elegans* qPCR assays is provided in the Supplementary Table S6.

### Western blotting

Cell pellets were lysed at a density of 1 × 10^6^ cells in lysis buffer [10 mM Tris–HCl (pH 7.5), 150 mM NaCl, 1 mM EGTA, 1% NP-40, 0.2% NaDeoxycholate, 1 mM DTT] supplemented with Complete Cocktail tablets (Roche), incubated for 20 min on ice and cleared by centrifugation. In all, 50 µg of proteins were separated by SDS–PAGE and probed with the indicated antibodies. Rabbit polyclonal anti-sera were raised against DHX34 and NBAS peptides and affinity purified (Eurogentec). Goat polyclonal anti-UPF1 (RENT1 Antibody, Bethyl, A300–038A) was used at a dilution 1:3000, and mouse monoclonal anti-β-tubulin (Clone TUB2.1, Sigma, T4026) was used at a dilution 1:5000. Proteins were visualized by ECL (Pierce).

## RESULTS

### Genome-wide identification of endogenous RNA targets regulated by DHX34 and NBAS in human cells, zebrafish embryos and *C. elegans*

DHX34 and NBAS function in the NMD pathway in nematodes and also in vertebrates; however, mechanistic aspects of their involvement in NMD await further investigation. Interestingly, despite NMD not being essential in *C. elegans*, both *smgl-1* (*NBAS*) and *smgl-2* (*DHX34*) were shown to be required for viability in nematodes (32). This strongly suggested that these two genes are likely to be involved in additional functions, other than NMD, which would render them essential in nematodes. NBAS, initially identified as a gene that is co-amplified together with *N-MYC* in human neuroblastomas (33), was recently reported to encode a peripheral membrane protein that is a component of the Syntaxin 18 complex, which has a role in Golgi-to-endoplasmic reticulum retrograde transport (51). By contrast, there are no published reports of the cellular roles for DHX34, apart from its proposed role in NMD.

We sought to identify the cellular RNAs that are regulated by DHX34 and NBAS in two vertebrate systems, human HeLa cells and the zebrafish, Danio rerio, as well as in the nematode *C. elegans*. For this, we used NimbleGen Gene Expression Arrays to profile changes in gene expression upon depletion of DHX34 or NBAS in these three species, using the core NMD factor UPF1 for comparison. Table 1 summarizes the expression array data in the three species studied. In HeLa cells, we used specific shRNAs that resulted in a efficient depletion of each protein (Supplementary Figure S1). Depletion of UPF1 affected the expression of 1336 genes at least 1.5-fold (∼10% of 14 089 expressed genes) (Table 1 and Supplementary Table S1), with most target genes being upregulated, confirming the role of UPF1 as a core NMD factor. Comparison with a previous study reporting that UPF1 regulates expression of diverse classes of mammalian transcripts (40) revealed a significant overlap of 46 regulated genes (*P*-value of 0.02). Moreover, 282 genes identified as UPF1 targets in a recent study by Yepiskoposyan and colleagues (44) were also present in our list of UPF1 regulated genes (*P* < 2.2e–16). A similar scenario was observed upon depletion of DHX34 where the proportion of upregulated genes is similar to the one observed on UPF1 knockdown. By contrast, depletion of NBAS altered the expression of 1444 genes, corresponding to ∼10% of expressed genes. However, the majority of NBAS targets were downregulated, which could suggest that this gene also plays a prominent role in another process distinct from NMD.

**Table 1.** Summary of microarray data in human cells, zebrafish and *C. elegans*

*Human* array: 27 063 genes			
Expressed genes: 14 089			

	Upf1 k.d.	DHX34 k.d.	NAG k.d.

All	1336	345	1444
Up	882	234	590
Down	454	111	854
Regulated (%)	10	3	10

Zebrafish array: 29 720 genes			
Expressed genes: 13 280			

	Upf1 k.d.	DHX34 k.d.	Nbas k.d.

All	1323	1475	3644
Up	1125	1209	1974
Down	198	226	1670
Regulated (%)	10	11	27

*C. elegans* array: 20 043 genes			
Expressed genes: 15 005			

	Upf1 k.d. (*smg-2*)	DHX34 k.d. (*smgl-2*)	NAG k.d. (*smgl-1*)

All	2049	584	3020
Up	1411	547	1962
Down	638	37	1058
Regulated (%)	14	4	20

Our previous work established that depletions of Upf1, Dhx34 and Nbas in zebrafish resulted in a specific defect in embryonic development, demonstrating the requirement of NMD for proper embryogenesis (37). For each gene, the specificity of knockdown-mediated phenotype was demonstrated by the use of both translation start-site targeting and splice-site targeting MOs, which induced identical phenotypes. The specificity of MO-induced phenotype was further validated by injections of corresponding 5 bp mismatch MOs for each targeted gene that resulted in a wild-type phenotype, to rule out off-target effects. To identify RNA targets regulated by NMD factors in zebrafish, one-cell-stage embryos were injected with MO targeting translation start sites of *upf1*, *dhx34* and *nbas* genes. Pools of ∼50 embryos from two independent injection experiments were compared with mock-injected control embryos. Here, depletion of individual NMD factors resulted in misregulation of a large number of genes (ranging from 10 to 27% of the genes expressed in embryos), inducing upregulation of a large majority of RNA targets (Table 1 and Supplementary Table S1). We used a similar approach in *C. elegans*, where we depleted NMD factors by feeding-mediated RNAi. Here, depletion of individual NMD factors affected ∼4–20% of expressed genes, and, again, a majority of RNA targets showed a marked upregulation (Table 1 and Supplementary Table S1). This experiment provides a comprehensive list of cellular RNAs whose expression is regulated directly or indirectly by DHX34 and NBAS in three different species (Supplementary Table S1).

### DHX34 and NBAS, together with UPF1, co-regulate a set of common RNA targets in three different organisms

We clearly observed that DHX34, NBAS and the core NMD factor UPF1 co-regulate a significant group of mRNA transcripts in all three species (Figure 1). In human cells, ∼47% of genes that were upregulated on DHX34 depletion were also upregulated by either UPF1 or NBAS (Figure 1A). In zebrafish, the proportion of DHX34 upregulated targets that behaved similarly upon depletion of UPF1 or NBAS increased to 87% (Figure 1B), whereas in *C. elegans*, ∼70% of *smgl-2* upregulated targets were also upregulated by *smg-2* or *smgl-1* (Figure 1C). A significant positive correlation between the changes in expression of target genes for both DHX34 and NBAS relative to UPF1 (*P*-value of 0.02 and 0, respectively) was observed in human cells (Figure 2A). In zebrafish, the majority of significantly regulated targets also show positive linear co-regulation between *upf1* and *dhx34* or *nbas*, respectively (*P*-values of 0 for both scatterplots) (Figure 2B). In *C. elegans*, the majority of target genes regulated by *smgl-2* or *smgl-1* showed the same trend of either up- or downregulation of expression as *smg-2* targets (*P*-value 0 for bot scatterplots) (Figure 2C). We noticed that the co-regulation observed with UPF1 in all three species could also be extended to UPF2 and SMG1 in human cells, suggesting that both DHX34 and NBAS do have a role in the NMD pathway (Supplementary Figure S2).

**Figure 3.**
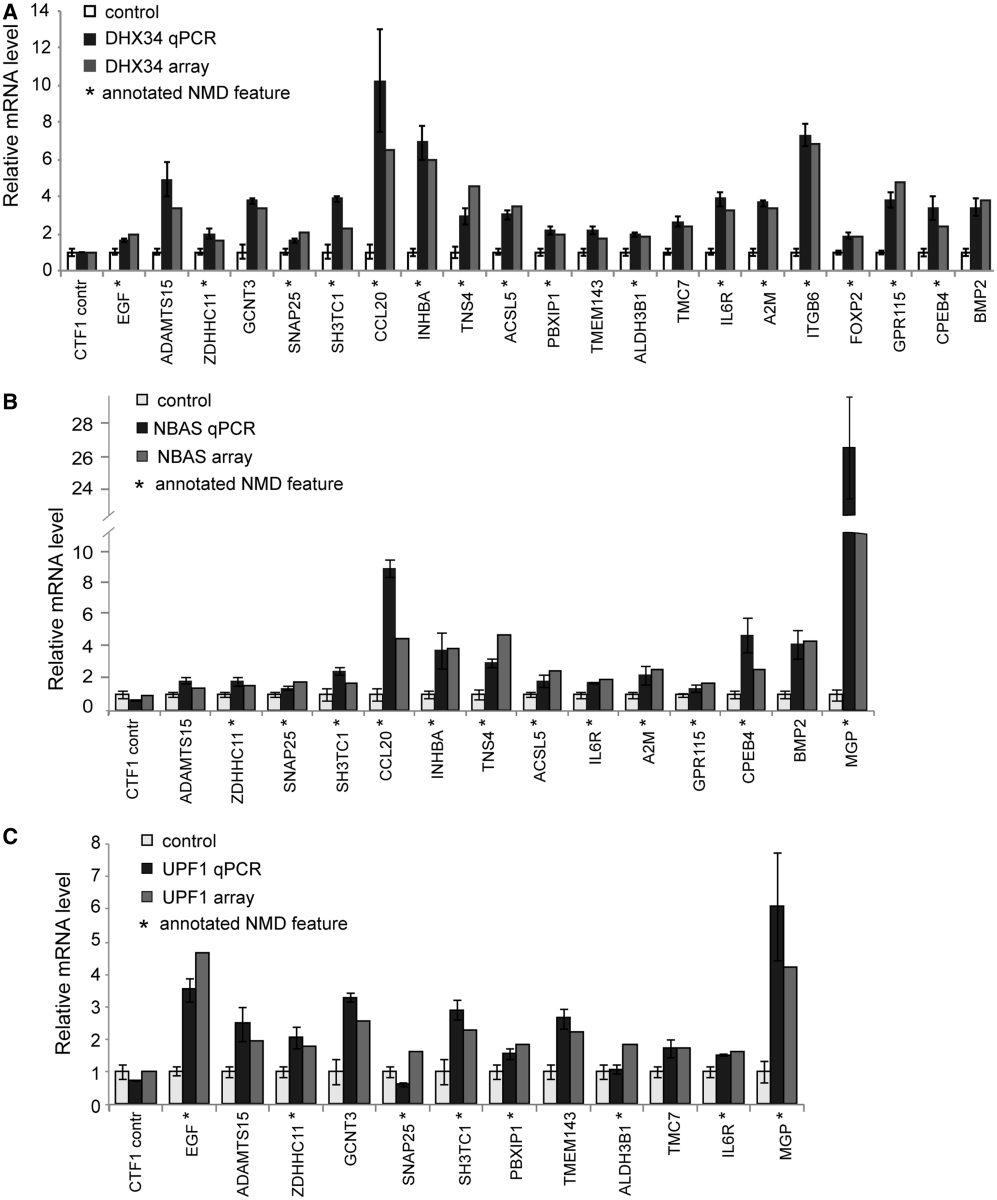
Validation of targets identified in the human microarrays. RNA levels of selected targets of (**A**) DHX34, (**B**) NBAS and (**C**) UPF1 were analyzed by RT-qPCR (black bars) and compared with the microarray data (gray bars) in HeLa cells depleted of the respective factors. In all panels, RNA levels of target genes in mock-depleted HeLa cells were used as a control (white bars). RT-qPCR expression values were normalized against three reference genes with constant expression, and values in control samples were set to one. Matrix GLA protein (MGP) was selected due to its marked upregulation upon NBAS knockdown in the microarray analysis. CTF1 (cardiotrophin 1) was chosen as a control due to its constant expression level in all data sets (Supplementary Table S6). An asterisk next to the gene name indicates the presence of an EnsEMBL-annotated NMD feature.

We selected a subset of 21 genes that were upregulated on DHX34 depletion in the human microarray data, most of which also showed co-regulation with NBAS and UPF1 (list of validated targets together with predicted NMD features is included in Supplementary Table S2). We used RT-qPCR to validate their expression upon depletion of DHX34, NBAS or UPF1 in HeLa cells (Figure 3A–C, respectively). In the case of DHX34, we observed upregulation of all these genes by RT-qPCR analysis, in good correlation with the microarray data (Figure 3A). A similar analysis was carried out with this subset of targets to validate their upregulation upon depletion of NBAS and/or UPF1 as seen in the microarray data. Here, again, we observe a good correlation between the microarray data and the RT-qPCR validation (Supplementary Table S2 and Figure 3B and C). In all cases, a majority of these regulated targets harbor NMD features. We also validated a small subset of zebrafish and *C. elegans* target genes that showed coregulation in the microarray data. We confirmed that in zebrafish four/five (80%) of target genes were specifically upregulated on MO-mediated depletion (Supplementary Figure S3A–C). In *C. elegans*, all tested targets showed upregulation on RNAi-mediated depletion of *smg-2* and *smgl-1*, and *smgl-2* knockdown resulted in upregulation of four/five (80%) of tested targets (Supplementary Figure S4).

### Analysis of DHX34 and NBAS targets

**Figure 4.**
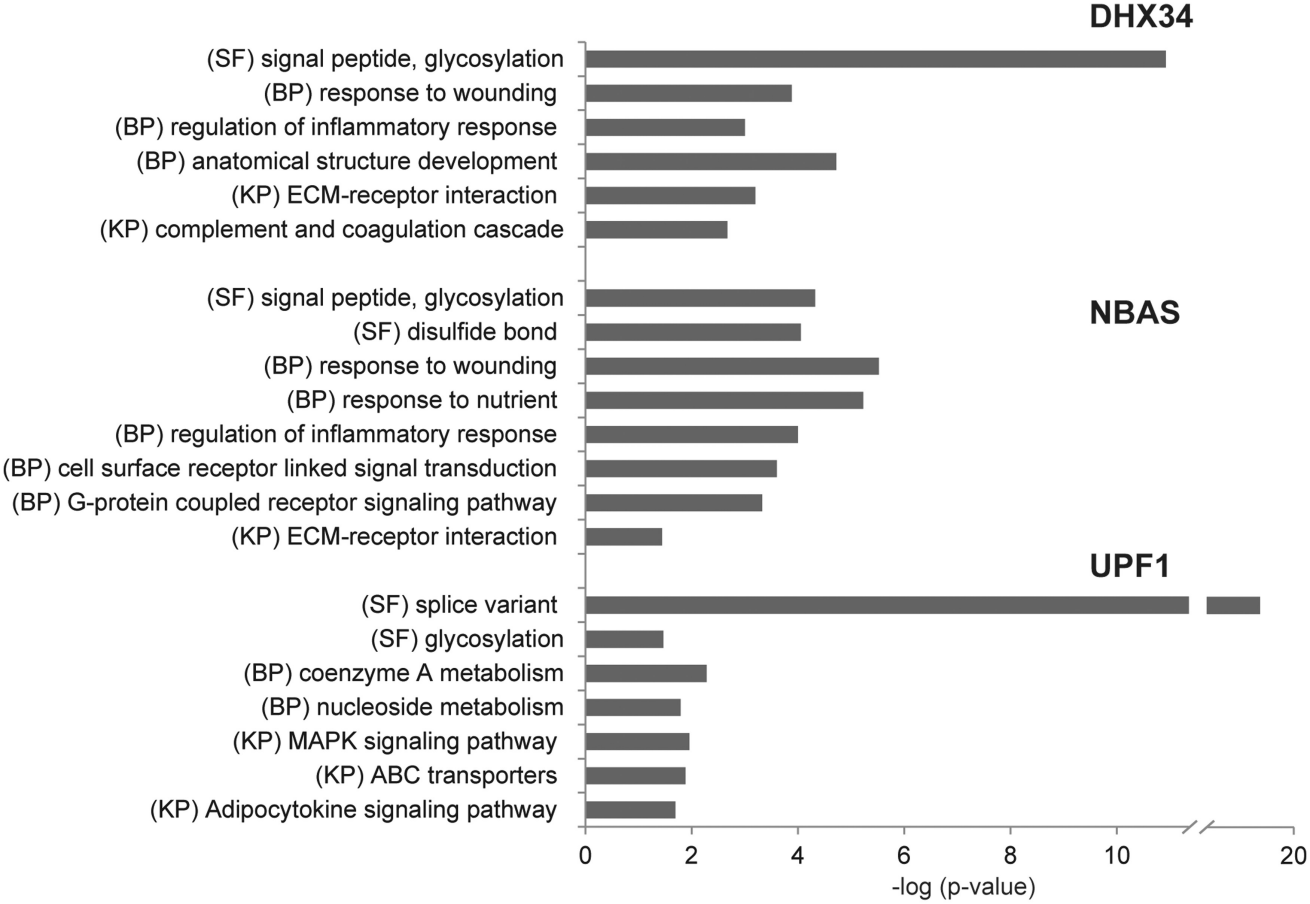
NMD regulates the expression of genes associated with cellular stress response pathways and membrane trafficking. Functional categories that are significantly enriched within DHX34, NBAS and UPF1 upregulated genes (relative to all expressed genes) are indicated. Functional categories analyzed were Sequence Feature (SF), GO Biological Process (BP) and KEGG Pathway (KP).

GO analysis of the target genes for both DHX34 and NBAS revealed that they regulate common pathways in human cells, zebrafish and *C. elegans*. Strikingly, functional categories describing sequence features associated with protein trafficking and ER-associated protein modifications were among those most significantly enriched for all target groups in human, zebrafish and *C. elegans*. In addition, GO terms and KEGG pathways describing cellular response to stress were also significantly enriched in all data sets for upregulated targets (Figure 4 and Supplementary Table S3). In zebrafish, additional categories that were significantly enriched are related to embryonic development, thus providing a link between NMD and the observed abnormal embryonic development phenotype upon depletion of Dhx34 and Nbas (37) (Supplementary Table S3). In *C. elegans*, additional functional categories associated with regulated genes are involved in lipid and polysaccharide metabolic processes (Supplementary Table S3). Interestingly, in human, a vast majority of NBAS regulated genes harbor sequence features associated with protein trafficking and ER-coupled protein modifications (*P*-value 1.8e–25) (Supplementary Table S3). This observation is intriguing, given the previously described role of NBAS in Golgi-to-ER retrograde transport and glycosylation of secretory proteins (51). Recently, a single point mutation in the *NBAS* gene was shown to cause a human syndrome with short stature, facial dysmorphism, optic atrophy and leucocytes anomaly, termed, SOPH (52). We have therefore looked for NBAS-regulated genes that could suggest a link with the SOPH syndrome. Strikingly, we observed that a significant number of NBAS targets were associated with GO terms describing bone mineralization and development and regulation of cholesterol biosynthesis, previously implicated with lymphocyte anomaly (Supplementary Table S4). Interestingly, the target that shows the strongest upregulation on NBAS depletion, matrix Gla protein (MGP) gene, acts as an inhibitor of bone formation. Defects in this gene are linked to Keutel syndrome that shares abnormal cartilage calcification phenotype (53).

### DHX34 and NBAS participate in an NMD negative feedback loop controlling the expression of NMD factors mRNAs

**Figure 5.**
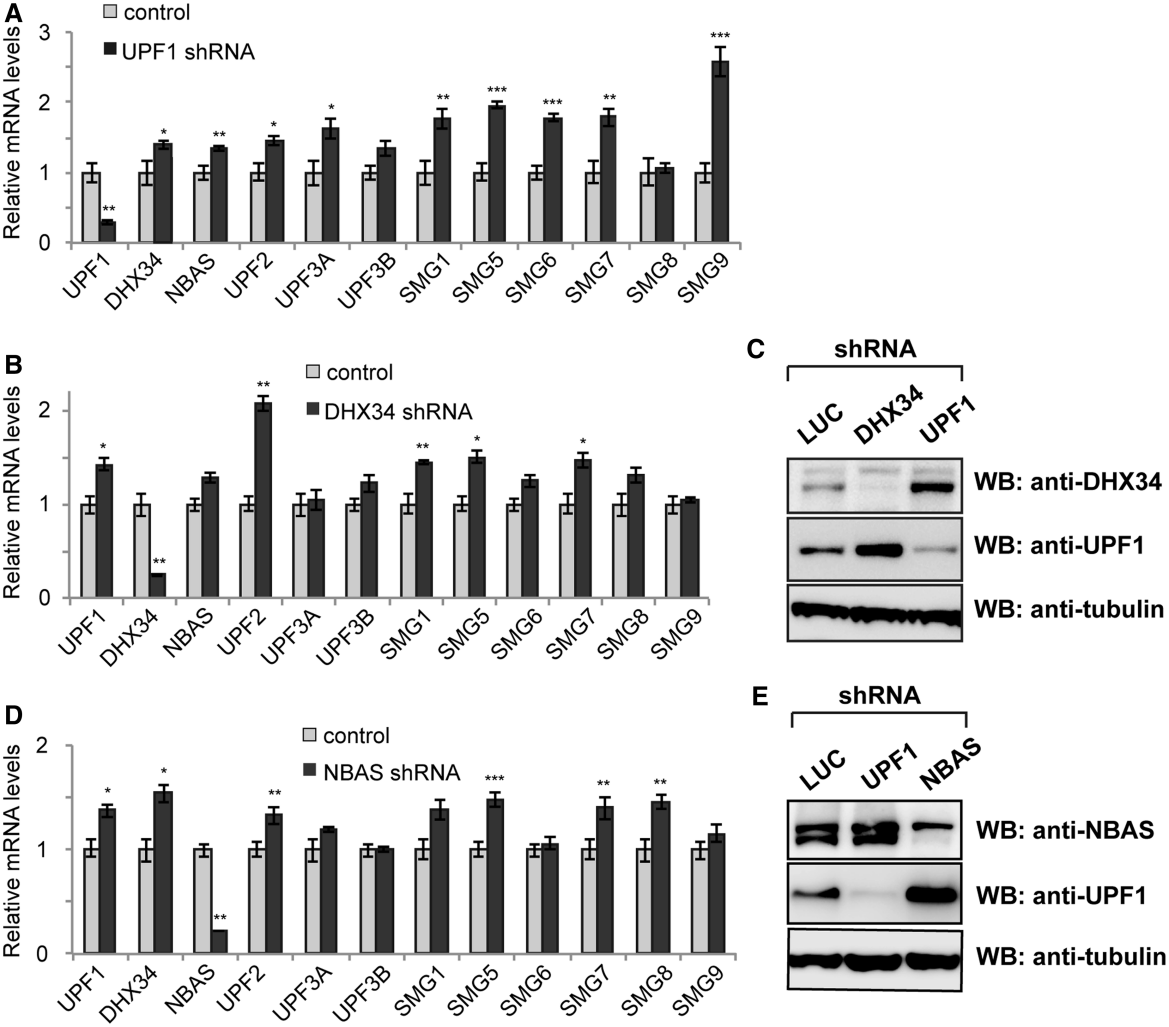
Autoregulation of the NMD pathway in human cells. DHX34 and NBAS contribute to the negative feedback-loop regulating levels of transcripts that encode NMD factors. RT-qPCR analysis of total cellular RNA from HeLa cells depleted of (**A**) UPF1, (**B**) DHX34 and (**D**) NBAS. The values shown are the average fold-change (mean ± SEM) from three independent experiments relative to mock-depleted cells (control). Statistical analysis was performed using the Student’s *t-*test. *P < 0.05; **P < 0.01; ***P < 0.001. (**C**) and (**E**) Western blot analysis shows the upregulation of UPF1 at the protein level upon depletion of DHX34 or NBAS, respectively, relative to mock-depleted cells (LUC, shRNA against luciferase transcript).

**Figure 6.**
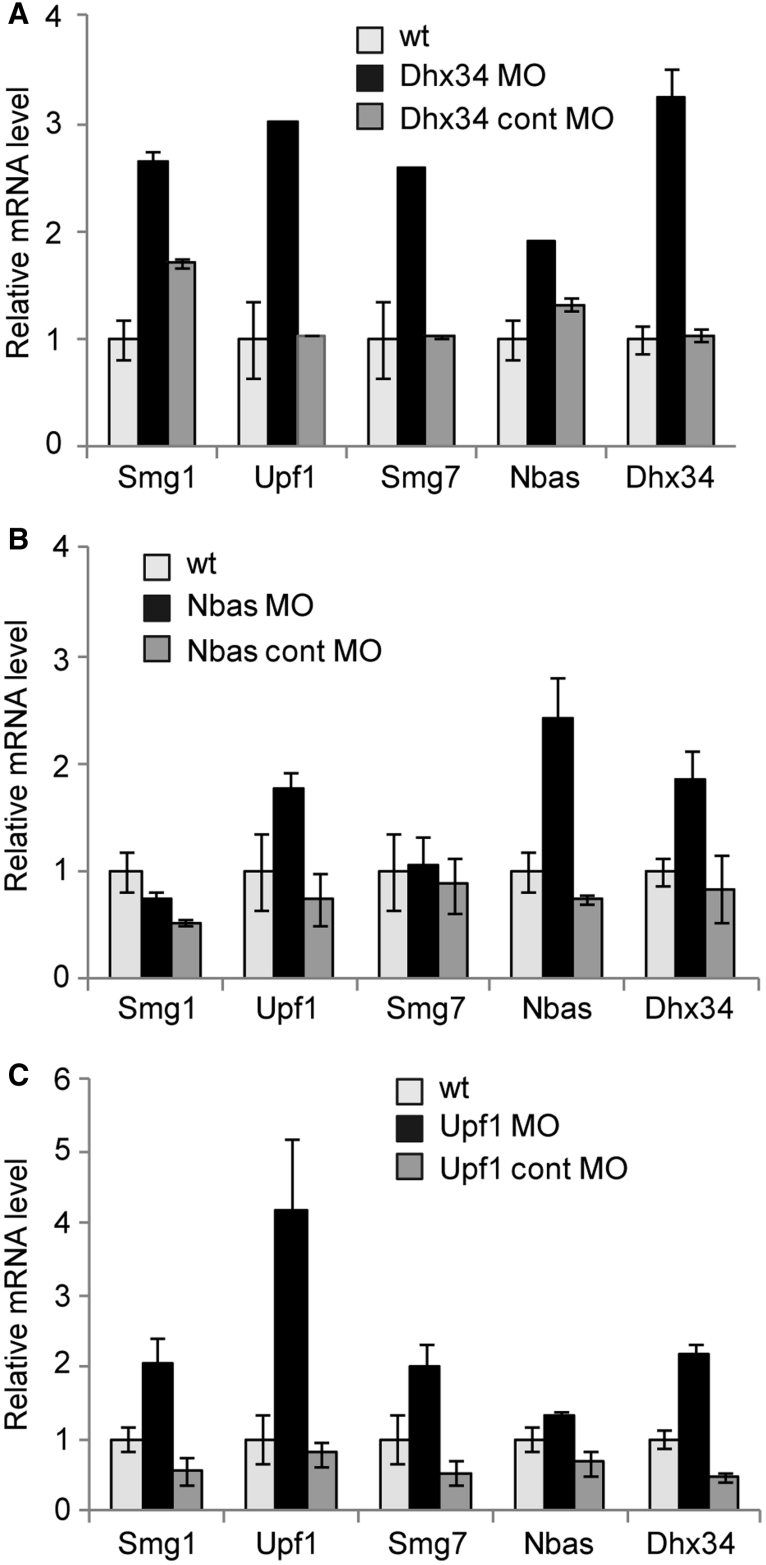
The NMD negative feedback loop is conserved in *Danio rerio*. Zebrafish embryos were injected with MO targeting Dhx34 (**A**), Nbas (**B**) or Upf1 (**C**). As a specificity control for each gene, a 5 bp mismatch MO (cont MO) was also injected. The levels of zebrafish NMD factor mRNAs were analyzed by RT-qPCR relative to a control. The values represent an average of three independent injection experiments (mean ± SEM). Because the MOs affect only the protein level of targeted genes, the upregulation of targeted gene transcripts is a further evidence for a feedback loop regulation.

The existence of a negative feedback regulatory network that directly acts on core NMD factors was initially reported in mammalian cells (43,44). This mechanism operates to maintain homeostasis when externals signals perturb the NMD response. We investigated whether abrogation of the expression of either DHX34 or NBAS would affect the levels of transcripts encoding NMD factors as was previously described for the depletion of core NMD proteins. Interestingly, we found that mRNAs encoding NMD factors were sensitive to depletion of either DHX34 or NBAS in all three species studied (Figures 5 and 6 and Supplementary Figure S6). For instance, UPF1 depletion in human cells resulted in marked upregulation of SMG1, SMG5, SMG6 and particularly SMG9 (Figure 5A), in agreement with previous reports (43,44). In the case of DHX34 depletion, a drastic upregulation of UPF2 was observed alongside increased mRNA levels for UPF1, SMG1, SMG5 and SMG7 (Figure 5B). Finally, depletion of NBAS resulted in a significant increase in mRNA levels of the core NMD factors UPF1, UPF2, SMG5, SMG7 and SMG8, together with a significant upregulation of DHX34 mRNA (Figure 5C). Previous work established that many NMD factors transcripts have NMD-inducing features such as long 3′untranslated regions (UTRs) or upstream open-reading frames (uORFs) (44). We therefore analyzed the transcripts coding for DHX34 and NBAS for putative NMD-inducing features. Our analysis revealed that the *DHX34* gene harbors five predicted uORFs, with the longest one coding for a 32 amino acid-long peptide (data not shown). A coupling of alternative splicing and NMD has been extensively studied in several organisms, including *C. elegans*, *Drosophila* and mammals (54–57) [reviewed by (58,59)]. Notably, the *DHX34* gene also encodes several transcripts where exon skipping gives rise to a PTC. Similarly, the NBAS gene encodes transcripts with alternative splicing-generated PTCs. Importantly, we confirmed that depletion of either DHX34 or NBAS results in the upregulation of UPF1 at the protein level (Figure 5C and E). To determine whether the UPF1 transcript is a direct target of DHX34 and NBAS, we performed an RNA half-life analysis of UPF1 mRNA levels on DHX34 and NBAS depletion. We observed a marked stabilization of UPF1 mRNA in both cases (Supplementary Figure S5A and B, respectively). This clearly establishes that both DHX34 and NBAS directly regulate the level of UPF1 mRNA and participate in the conserved NMD negative feedback regulatory loop, as was described for core NMD factors in mammalian cells (43,44).

### The NMD negative feedback loop is conserved throughout evolution

Interestingly, we noticed that the upregulation of transcripts encoding NMD factors in response to NMD abrogation was also evident in both zebrafish and in *C. elegans*, indicating that the NMD autoregulatory loop is conserved through evolution (Figure 6 and Supplementary Figure S6). In zebrafish, morpholino-mediated knockdown of Dhx34 significantly upregulated the levels of all tested mRNAs encoding NMD factors (Figure 6A), whereas depletion of NBAS led to a more selective upregulation of *upf1*, *nbas* and *dhx*34 mRNAs (Figure 6B). As expected from studies in mammalian systems, Upf1 depletion resulted in an upregulation of the majority of mRNAs encoding NMD factors (Figure 6C). Intriguingly, in each case, we observed the strongest upregulation of the mRNA encoding for the NMD factor that we were depleting. We speculate that because the MOs prevent the production of targeted protein without affecting the mRNA level, the upregulation of the corresponding transcript is a further indication in support of a feedback loop regulation. A similar scenario was observed in *C. elegans*, where *smg-2* depletion results in a marked increase of *smg-1* and *smgl-1* mRNAs (Supplementary Figure S6). Similarly, a significant increase in *smg-1* mRNA was also observed upon depletion of *smgl-1*. These data indicate that NMD homeostasis is regulated via a negative feedback loop in zebrafish and in *C. elegans*, suggesting that this regulatory mechanism is potentially present in other species where NMD is conserved.

## DISCUSSION

Here, we provide a comprehensive list of endogenous cellular RNA targets regulated by DHX34 and NBAS in human cells, zebrafish and *C. elegans* (Supplementary Table S1). We found a large number of genes that are upregulated upon depletion of each of these NMD factors in the three organisms, and these could be interpreted to be direct NMD targets. We also observed a large proportion of genes that were downregulated upon depletion of individual factors, in particular in the case of NBAS. This is not uncommon and most likely includes either indirect NMD targets or genes that are regulated by the factor under study but independently of NMD. A large number of UPF1-regulated targets were not significantly affected by the depletion of DHX34 or NBAS (Figure 1). This is not unexpected, taking into consideration the reported non-NMD functions of UPF1, such as Staufen-mediated decay, telomere maintenance, DNA replication and translation (60–62). Similarly, DHX34 and NBAS are also likely to perform additional, NMD unrelated roles, as they are the only NMD factors that are essential in *C. elegans* (32). Furthermore, the reported involvement of NBAS in ER-Golgi transport may account for the large proportion of targets that are downregulated in response to NBAS depletion (51). It has been proposed that in mammals, there are several alternative NMD branches that differ in their requirement of NMD factors. For example, alternative NMD branches that are UPF2, core EJC or UPF3-independent but UPF1-dependent have been described previously (63–65). Therefore, another group of UPF1-unique targets would consist of those targets that do not require DHX34 or NBAS to undergo NMD. Thus, the existence of target genes that are regulated by UPF1 but not by DHX34 or NBAS can be explained by non-NMD functions of UPF1 or alternatively by DHX34/NBAS-independent branches of the NMD pathway.

Importantly, we clearly observed that DHX34, NBAS and the core NMD factor UPF1 co-regulate a significant group of mRNA transcripts in each organism (Figure 1). This suggests that DHX34 and NBAS act in concert with core NMD factors to co-regulate a significant number of endogenous RNA targets. Our analysis also revealed that there are few conserved targets for DHX34 and NBAS across the three species, which is not entirely surprising, given the evolutionary distance between these organisms. However, we identified groups of targets that are predicted to function in similar processes that are conserved across human, zebrafish and *C. elegans*, with the most commonly affected process being the response to cellular or environmental stress (Figure 4). Thus, despite the lack of conservation of individual NMD targets, there is a significant conservation in the cellular pathways that are regulated by the NMD response across different organisms. Previous work clearly established that the NMD process is itself inhibited by various cellular stresses. For example, amino acid deprivation was shown to cause inhibition of NMD that in turn led to upregulation of genes governing the amino acid homeostasis (40). Similarly, cellular stresses such as hypoxia or the unfolded protein response that resulted in the phosphorylation of eIF2α, also inhibit NMD (66,67). Here, we show that NMD modulates the expression of transcripts that are involved in the regulation of cellular stress response in an evolutionary conserved manner. Thus, the physiological role of NMD is broadly conserved across evolution and can be seen as part of a conserved feedback loop that helps the organism to respond to the environmental and cellular stress.

Of particular interest, a mutation in the *NBAS* gene has been reported in a hereditary short stature syndrome (SOPH syndrome) in the Yakuts population that lives in the far east of the Russian Federation. This autosomal recessive disorder is characterized not only by short stature but is also associated with optic nerve atrophy, postnatal growth retardation, facial dysmorphism and Pelger–Huët anomaly of leucocytes (52). The single amino acid substitution does not seem to affect the expression of the protein but may compromise its activity. It is tempting to speculate that lack of function of NBAS could affect its role in NMD, its activity as a component of the syntaxin 18 complex or compromise a yet unreported cellular function. Interestingly, among the targets regulated by NBAS, there are several genes that have a role in bone development and cholesterol biosynthesis. Defect in these processes could help to explain the SOPH phenotype. In particular, depletion of NBAS led to a significant upregulation of MGP that regulates bone formation. Thus, an increased MGP activity would be compatible with a described short stature and associated bone defects present in the SOPH syndrome.

The feedback mechanism was shown to act in different branches of the NMD pathway in a cell-type-specific and developmentally regulated manner. Of importance, we established that not only DHX34 and NBAS co-regulate target genes with core NMD factors but also participate in a negative feedback regulatory loop that acts to tightly regulate the NMD response. The existence of such a negative feedback regulatory network that targets core NMD factors, as well as DHX34 and NBAS themselves, acts to maintain homeostasis when the NMD response is perturbed by external signals, such as stress (43,44,67). A tight control of the NMD response is not only provided by the feedback loop described earlier in the text, whereby mRNAs encoding NMD factors are themselves targets of the NMD response. It was recently elucidated that a brain-specific microRNA, miR-128, targets UPF1 and the exon-junction complex core component MLN51 in neural cells, negatively regulating their expression and reducing the NMD response. This mechanism particularly operates in differentiating neuronal cells and during brain development. This results in the upregulation of mRNAs that are normally targeted for decay by the NMD pathway (68). As discussed a variety of cellular stresses result in downregulation of the NMD response (66,67). As such, the efficiency of NMD has shown to be affected in different cellular contexts, such as cancer [for a recent review, see (67)]. It may well be that *DHX34* and/or *NBAS* mRNAs are regulated by the miRNA pathway, altering their relative levels in a tissue-specific or developmentally regulated manner or in response to cellular stress. Future studies will investigate how the expression of DHX34 and NBAS is regulated and its precise mechanism of action in the NMD pathway.

Altogether, these data show that both DHX34 and NBAS act in concert with core NMD factors to co-regulate a large number of endogenous RNA targets throughout evolution. The conservation of a mechanism to tightly control NMD homeostasis across different species highlights the importance of the NMD response in the control of gene expression.

## SUPPLEMENTARY DATA

Supplementary Data are available at NAR Online.

## FUNDING

Medical Research Council (to E.E.P., J.F.C.); Wellcome Trust [095518/B/11/Z to J.F.C.]; Wellcome Trust [082433/Z/07/Z to C.A., E.E.P.]. Funding for open access charge: Wellcome Trust [095518/B/11/Z].

*Conflict of interest statement*. None declared.

## Supplementary Material

Supplementary Data
