# Supplementary material for: DHX34 and NBAS form part of an autoregulatory NMD circuit that regulates endogenous RNA targets in human cells, zebrafish and Caenorhabditis elegans
Source: Nucleic Acids Res. 2013 Jul 4;41(17):8319–31. doi: 10.1093/nar/gkt585 (PMC3783168; doi:10.1093/nar/gkt585)
Supplement: Supplementary Data [file supp_41_17_8319__index.html]

DHX34 and NBAS form part of an autoregulatory NMD circuit that regulates endogenous RNA targets in human cells, zebrafish and Caenorhabditis elegans — DHX34 and NBAS form part of an autoregulatory NMD circuit that regulates endogenous RNA targets in human cells, zebrafish and Caenorhabditis elegans — Supplementary Data 

# DHX34 and NBAS form part of an autoregulatory NMD circuit that regulates endogenous RNA targets in human cells, zebrafish and *Caenorhabditis elegans*

## 

files

**Files in this Data Supplement:**

- Supplementary Data - pdf file
- Supplementary Data - xlsx file
- Supplementary Data - xlsx file
- Supplementary Data - xlsx file
- Supplementary Data - xlsx file
- Supplementary Data - xlsx file
