# Supplementary material for: DHX34 and NBAS form part of an autoregulatory NMD circuit that regulates endogenous RNA targets in human cells, zebrafish and Caenorhabditis elegans
Source: Nucleic Acids Res. 2013 Jul 4;41(17):8319–31. doi: 10.1093/nar/gkt585 (PMC3783168; doi:10.1093/nar/gkt585)
Supplement: Supplementary Data [file supp_gkt585_nar-00881-a-2013-File009.pdf]

SUPPLEMENTARY DATA

DHX34 and NBAS form part of an autoregulatory NMD circuit that regulates endogenous RNA targets in human cells, zebrafish and *C. elegans*

Dasa Longman, Nele Hug, Marianne Keith, E. Elizabeth Patton, Corina Anastasaki,[1] Graeme Grimes and Javier F. Cáceres[*]

Supplementary figures S1-S6

Supplementary Table 1. List of all targets for DHX34, NBAS and UPF1

Supplementary Table 2. List of human validated targets with NMD features

Supplementary Table 3. Target analysis

Supplementary Table 4. hsNBAS targets consistent with SOPH phenotype

Supplementary Table 5. List of morpholino oligonucleotides used in this study. Small letters indicate the position of the mismatches in the 5bp-mismatch control MOs.

Upf1 translation initiation site block:

Upf1 TB: 5’-CGCCTCCACACTCATCTTTATATTC-3’

Upf1 TBcont: 5’-CGgCTgCACAgTCATgTTTATtTTC-3’

Dhx34 Translation initiation site block:

Dhx34 TB: 5’-GACTCTCTTCATCCTTACGGGACAT-3’

Dhx34 TBcont: 5’-GAgTgTCTTgATCgTTACcGGACAT-3’

Nbas Translation initiation site block

Nbas TB: 5’-ATCACCTGCCATGTTTTCAATGAAC-3’

Nbas TBcont: 5’-ATCACgTcCgATcTTTTCAATcAAC-3’

Supplementary Table 6. List of human, zebrafish and *C.elegans* validated genes and corresponding qPCR assays

Supplementary Figure S1

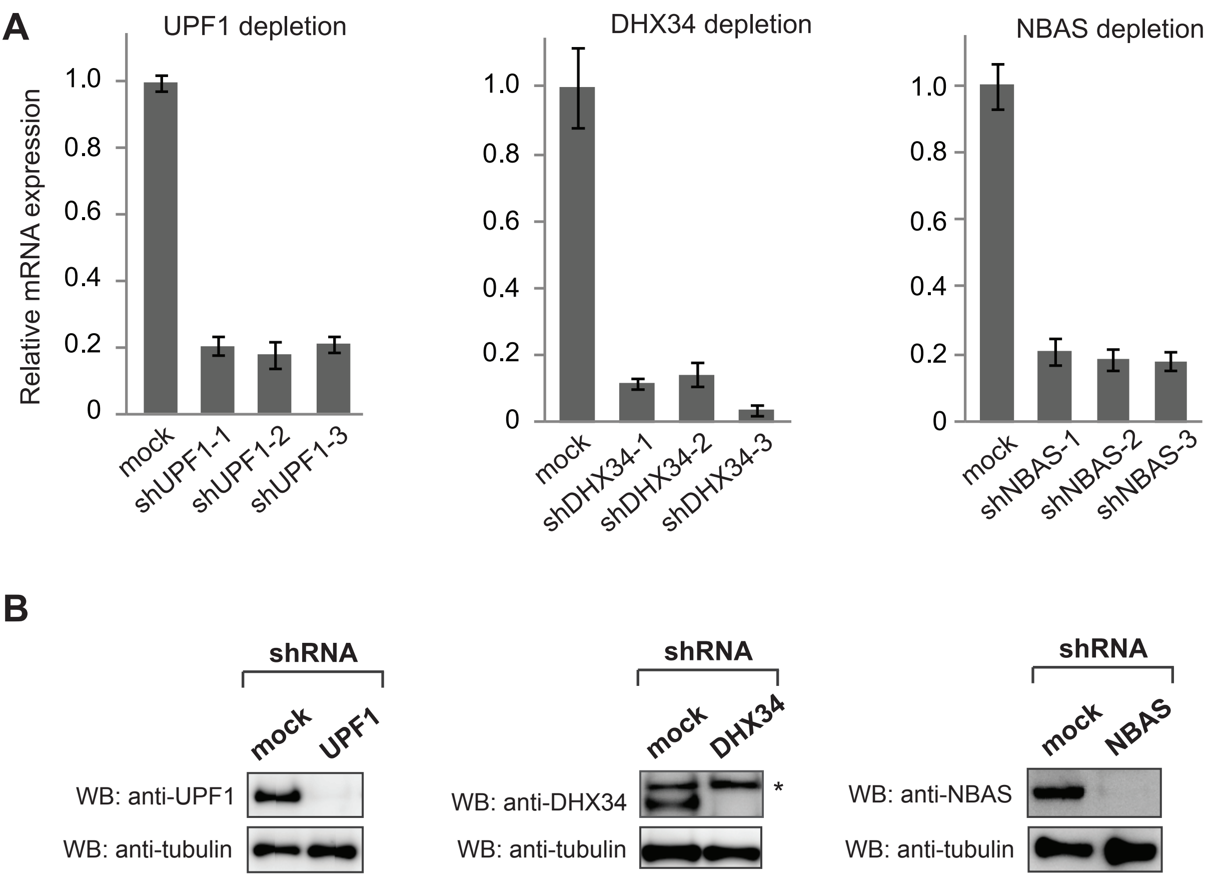

Figure S1. Depletion of UPF1, DHX34 and NBAS. (A) Levels of depletion of UPF1, DHX34 and NBAS mRNAs in HeLa cells as measured by RT-qPCR. Each depletion was performed in three biological replicas. (B) Representative Western blots measuring the depletion of UPF1, DHX34 and NBAS proteins upon knock-down with specific shRNAs. The asterisk denotes the presence of an unspecific band in the DHX34 panel.

Supplementary Figure S2

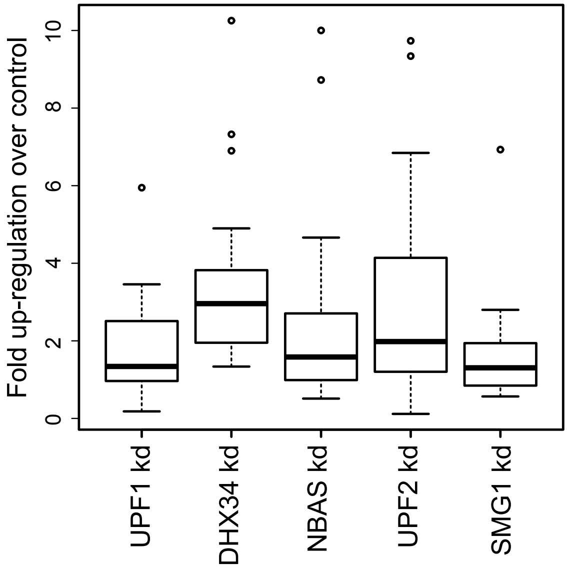

Figure S2. UPF2 and SMG1 regulate the expression of target genes in a manner similar to UPF1, DHX34 and NBAS in human cells. A box-plot shows the level of upregulation for selected target genes upon UPF1, DHX34, NBAS, UPF2 and SMG1 depletion. A panel of 22 genes described in Figure 3 was used for the analysis by RT-qPCR.

Supplementary Figure S3

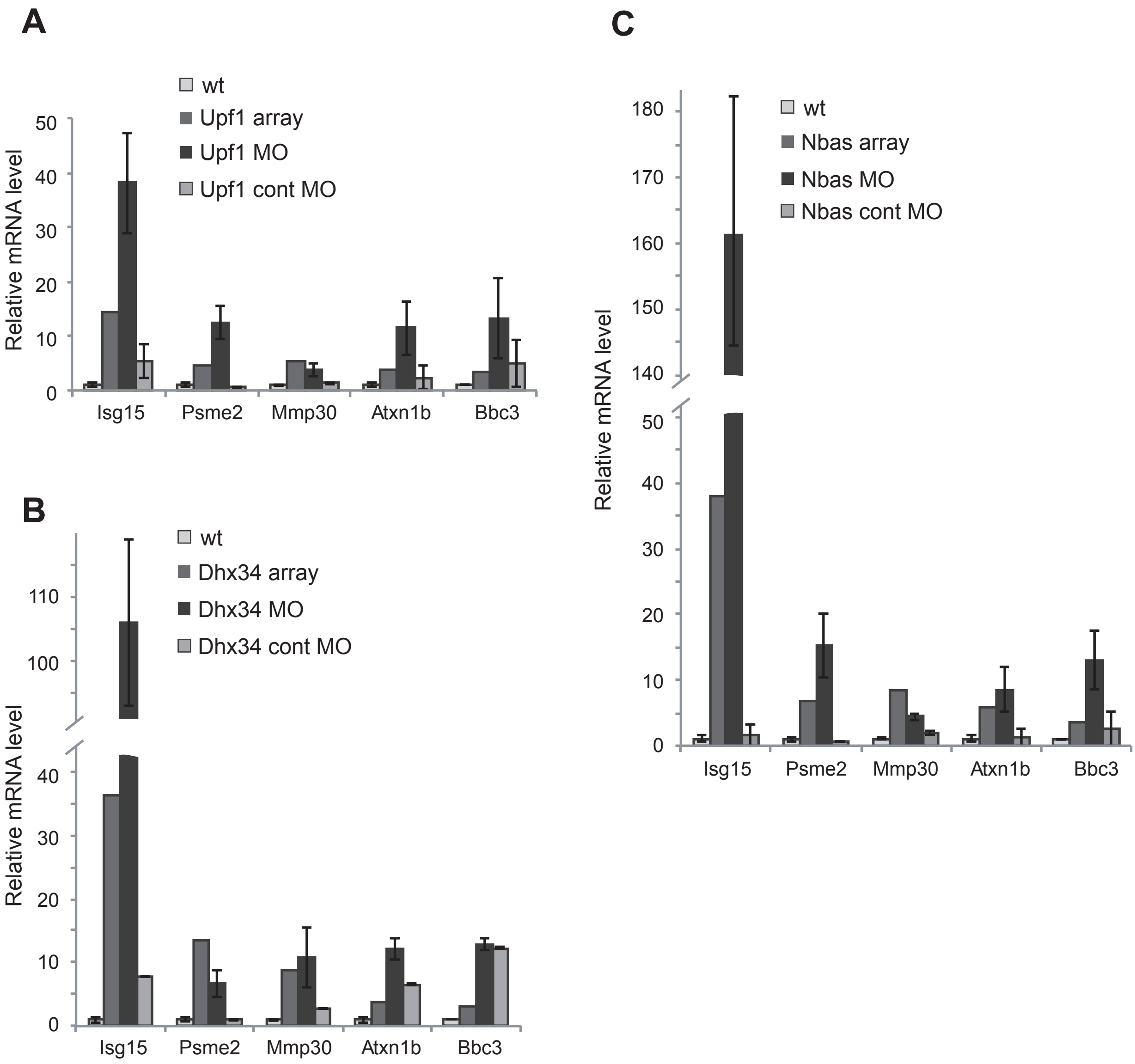

Figure S3. Validation of zebrafish NMD targets. Selected targets that were upregulated in all datasets were tested by RT-qPCR upon morpholino-induced depletion of (A) Upf1 (B) Dhx34 (C) Nbas. In each case depletion was achieved by injection of a gene-specific morpholino (MO). As a specificity control the effect of a 5bp mismatch control morpholino (cont MO) was analysed in parallel.

Supplementary Figure S4

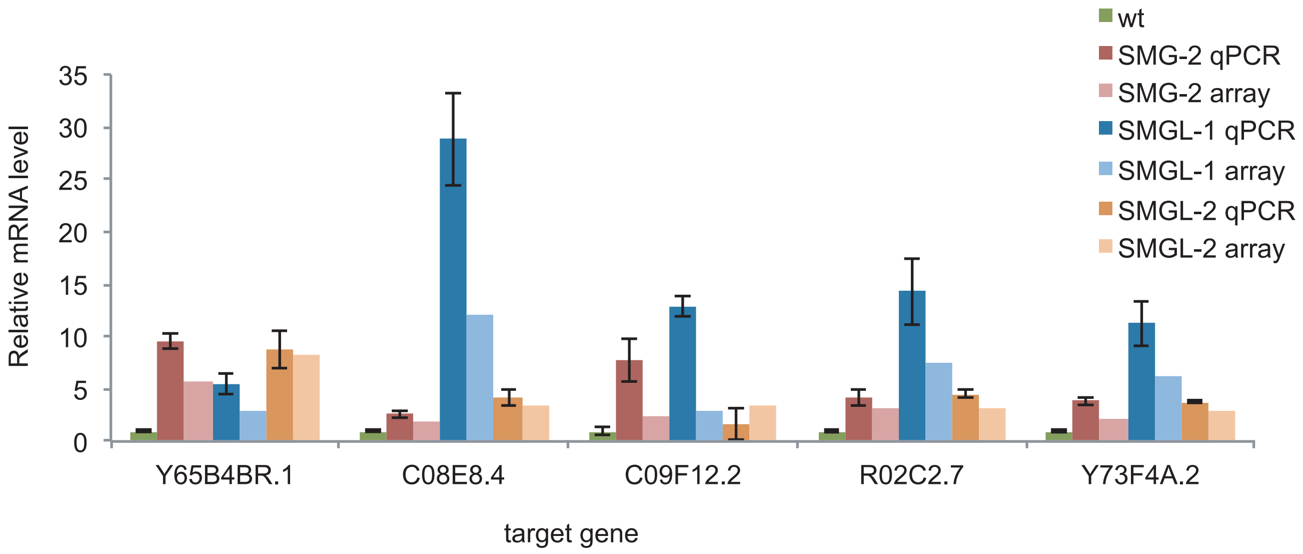

Figure S4. Validation of *C.elegans* NMD targets. Selected targets that were upregulated in all datasets were tested by RT-qPCR upon RNAi-induced depletion of SMG-2, SMGL-1 and SMGL-2. In each case depletion was achieved by microinjection of gene-specific dsRNA.

Supplementary Figure S5

A

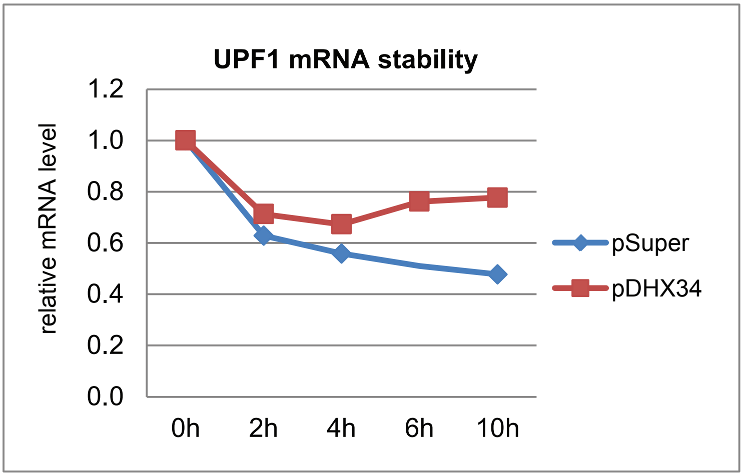

B

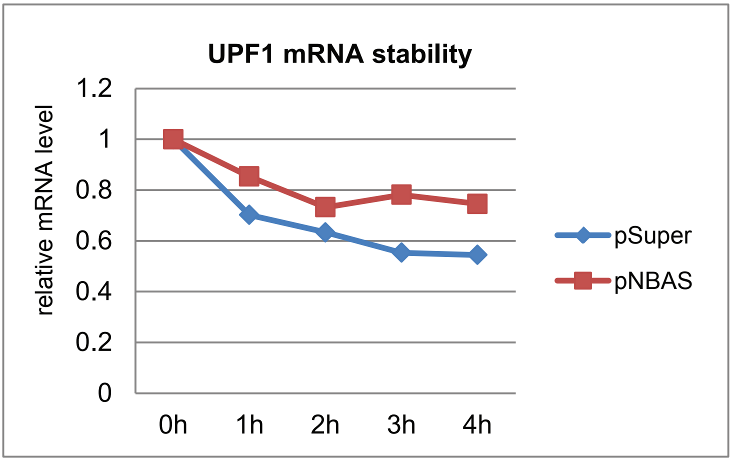

Figure S5. UPF1 transcript is stabilized upon knockdown of DHX34 (A) or NBAS (B). Hela cells were mock-depleted or depleted with DHX34 or NBAS shRNAs for 5 days and then treated with actinomycin D (5μ/ml) for indicated times.Total RNA was analyzed by RT-qPCR. The level of UPF1 mRNA was normalized to two reference genes (ACTB and POLR2J).

Supplementary Figure S6

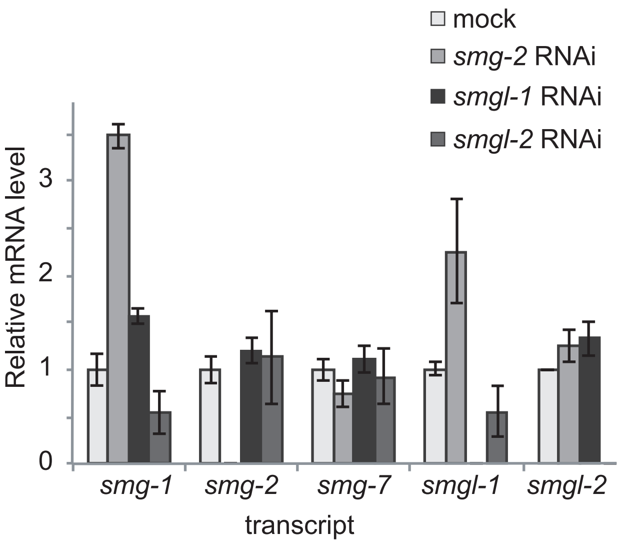

Figure S6. The NMD negative feedback loop is conserved in nematodes. *C.elegans smg-2*, *smgl-1* and *smgl-2* transcripts were depleted by feeding-mediated RNAi. The levels of NMD factor mRNAs were analyzed by RT-qPCR relative to the empty vector RNAi control (mock). The values represent an average of three independent experiments (mean±SEM). Since the plasmids used for the feeding-mediated RNAi contain gene-specific sequences it was not possible to use qPCR to quantify the transcript levels of depleted genes.
